# The Uneven Effect of Rare Diseases on Functional Status and Work Capacity

**DOI:** 10.3390/healthcare13060594

**Published:** 2025-03-08

**Authors:** Corina Oancea, Despina Mihaela Gherman, Florina Georgeta Popescu, Sorina Maria Aurelian, Corina Homentcovschi

**Affiliations:** 1 Faculty of Medicine, Carol Davila University of Medicine and Pharmacy Bucharest, 050474 Bucharest, Romania; corina.oancea@umfcd.ro (C.O.); despina.gherman@umfcd.ro (D.M.G.); corina.homentcovschi@umfcd.ro (C.H.); 2 Department of Occupational Health, Victor Babes University of Medicine and Pharmacy Timisoara, 300041 Timișoara, Romania

**Keywords:** rare diseases, functional impairment, risk factors, work capacity, quality of life

## Abstract

**Background:** Rare diseases are defined as clinical conditions that affect only a small number of persons in a population, considered fewer than 1 per 2000 in the European Union or fewer than 1 per 1600 in the United States They are serious, often chronic and progressive conditions, characterized by a pronounced clinical polymorphism that crosses all medical specialties. Multiple areas of life beyond just physical health are affected with significant impact on patients, families, and healthcare systems. **Objective:** To analyze the socio-demographic, medical, and vocational characteristics that correlate with functional status and work disability as a measure of quality of life in rare diseases. **Methods:** An observational retrospective study of adults with rare diseases evaluated for eligibility for social insurance rights in the National Institute of Medical Assessment and Work Capacity Rehabilitation Bucharest (INEMRCM, the Romanian abbreviation) over a 5-year period was made. Descriptive analysis was used to present sample characteristics. Means and standard deviations (SD) were calculated to describe numerical variables, frequencies were used to describe categorical variables, and logistic regression analysis was conducted to evaluate potential predictors of work capacity. All statistical analyses were performed by PSPP.3 software. *p* < 0.05 was the cut-off for statistical significance with a 95% confidence interval. **Results:** 90 consecutive persons were included in the survey. The mean age of the group was 44.5 years ± SD 10.61 years, with a female/male ratio of 48/42 persons. The mean disease duration was 10.61 years ± SD 9.76 years. Men had more severe disease (73.81%); *p* = 0.018 and significantly younger retirement age, M/F = 39.10 ± 12.26/43.06 ± 9.32; *p* = 0.037. Less disabling diseases were predominant autoimmune conditions (85.71% of cases); genetic conditions had a more severe functional impact in 63.75% of cases; *p* = 0.037. People with multisystem diseases but with specific or targeted treatment can work more frequently (76.19%); those with visual impairment have more severe impairments (73.77%); *p* < 0.001. All individuals who received specific therapy had a better functional status, unlike only 37.21% of those who received symptomatic treatment or treatment for complications; *p* = 0.023. Logistic regression analysis indicated that the type of impairment and the availability of specific treatments could serve as predictors of a reduced likelihood of employment in rare disease cases. Education level and occupation were not correlated with functional impairment and work disability (NS). **Conclusions:** Several factors, including some that are modifiable, were associated with better outcomes, such as reduced disability and an increased potential for work participation. Sex, disease etiology, type of impairment, and treatment were all significantly linked to functional capacity. Among these, the type of impairment and the availability of specific treatments might be predictors of employment. Addressing these parameters requires a multidisciplinary team, involving specialized care and comprehensive support services to improve the overall quality of life of individuals affected by rare diseases.

## 1. Background

Rare diseases are an emerging global public health concern, gaining increasing visibility due to their impact on the affecteds’ health, social life, and professional functioning. While these diseases are individually rare, they are collectively becoming more prevalent at a population level as they are identified more often due to advancements in genomic medicine. There are described between 6000 and 8000 rare diseases [1]. Epidemiological data indicate that 5–10% of the global population, or approximately 350–400 million people, are affected. Of these, 80% are genetic in origin, and 50% are children [1,2]. There are extremely rare infectious diseases, as well as autoimmune conditions and rare cancers. Currently, the cause of many rare diseases remains unknown. These conditions impact approximately 6–8% of the European population. While relevant epidemiological studies are lacking in Romania, it is reasonable to assume that European estimates apply, suggesting that at least 6% of the Romanian population is affected by a rare disease [3].

Rare diseases are defined by their low prevalence in the population, yet they represent a heterogeneous group, varying in etiology, onset, clinical manifestations, progression, and their effects on daily life and employment [4,5]. Their clinical pictures are characterized by a pronounced clinical polymorphism that crosses all medical specialties. There is great clinical variability between diagnoses and between patients with the same diagnosis. The management of patients with rare diseases is complex and involves multidisciplinary care and follow-up, including collaboration between national and international centers.

Late-onset genetic conditions or those in which affected children survive evolve into chronic disorders, exhibiting distinct characteristics from pediatric cases and leading to lifelong disabilities [6]. They significantly lower quality of life through physical and/or cognitive impairments, lead to capacity limitations, and/or participation restrictions in many domains of individual functioning, and are a leading cause of premature death. These concepts are based on ICF (International Classification of Functioning, Disability and Health) principles [7]. ICF, developed by the World Health Organization (WHO), provides a detailed framework to describe health-related aspects of personal performance.

Activity limitations refer to difficulties individuals may experience in executing specific tasks. They are mainly related to basic daily functions, are influenced by health conditions, and can range from mild to severe. These limitations affect psychological, emotional, and social well-being, influencing overall quality of life. They impact not only daily activities but also education, work, and social interactions, leading to participation restrictions.

Current medical practice is dominated by the biomedical model of health and disease. The model attributes a key role to biological determinants and explains disease as a condition caused by external pathogens or disturbances in the functioning of organs and body systems. However, the question of its effectiveness has recently been raised. It has become essential to expand the biomedical approach and to give equal weight to psychosocial factors to improve the effectiveness of disease treatment and control. It is now generally accepted that illness and health are the result of the interaction of biological, psychological, and social factors. According to the WHO constitution, health is defined as “the state of complete physical, mental and social well-being and not merely the absence of disease or infirmity” [8].

A diagnosis often disrupts the balance between personal and professional life. Many adult patients face the need to reduce or even cease professional activities and seek social security support.

The concept of work ability has constantly changed with time and further research. Traditional models focused on the medical aspects of health and functional capacity and on the importance of the balance between human resources and work demands, were replaced by the current bio-psycho-social model derived from ICF. Multidimensional models have highlighted, in addition to the traditional factors, other aspects that influence an individual’s health and work capacity, acknowledging the complex interplay of the work community, management, and the environment outside work life [9].

The evaluation of work capacity varies across countries, influenced by the structure of social security systems and specific legislation. Each EU member state has its own legal framework determining the entitlement to benefits, the calculation methods, the amount, the payment duration, and the minimum required work period to qualify for a benefit [10,11]. Thus, the evaluation processes and benefits across countries are not directly comparable and differ significantly. Nevertheless, numerous studies have shown that many people with various rare diseases (e.g., genetic conditions) face work-related challenges and have a high disability rate [12,13].

In Romania, current medical criteria classify work ability based on remaining functional capacity, categorizing it as either fully preserved or reduced. Residual functional capacity represents an individual’s ability to perform tasks despite functional limitations caused by a medical condition [14,15]. In cases of reduced capacity, individuals may still engage in professional activities under specific conditions. Depending on the assigned degree of work disability, they may also qualify for a work disability pension. This form of social protection, also known as an invalidity or long-term work incapacity pension, serves as a contributory, insured replacement income.

**Figure 1:**
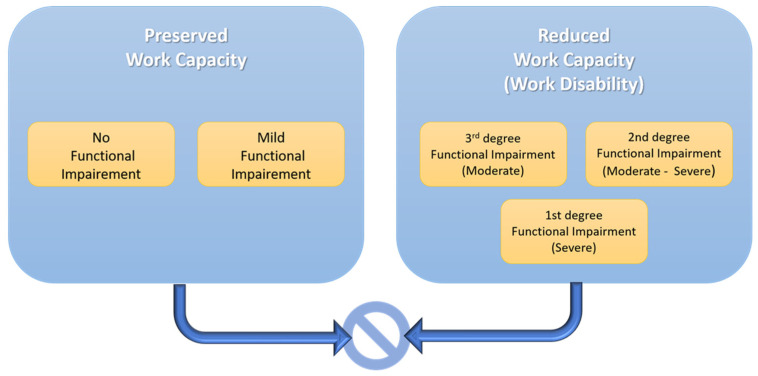
Representation of the opposing concepts of work capacity and work disability, emphasizing the impact of functional impairment on different levels of work disability.

Individual functional status (functional capacity) measures a person’s ability to carry out daily activities and tasks essential for independent living, employment, and social participation [16]. Work disability is assessed based on the extent of functional impairment. According to Law 360/2023 on the public pension system, disability is classified into three degrees:

- First degree: Severe functional impairment with a total loss of self-care, requiring daily assistance for basic activities.
- Second degree: Moderate-severe functional impairment with a partially preserved ability for self-care.
- Third degree: Moderate functional impairment with preserved self-care abilities (Figure 1) [17].

Similar classifications of reduced work capacity exist in other countries, such as the Czech Republic, Bulgaria, Cyprus, and Latvia [11].

Patients, particularly those with rare diseases, may be entitled to a second type of social assistance, a monthly disability allowance. This benefit is provided regardless of income or prior social insurance coverage, is governed by a different legal framework, assessed by a separate entity, and financed from a distinct fund. The recognition of disability is conducted in accordance with Law No. 448/2006, including its subsequent updates and amendments, on the protection and promotion of the rights of persons with disabilities. Additionally, individuals recognized as disabled may also access other social services, such as free healthcare and treatment, tax exemptions, transport benefits (e.g., free parking), and other facilities [18]. In other countries (e.g., Belgium, Czech Republic), compensation for incapacity for work and disability are also established as separate branches of the insurance system [11].

There are many other difficulties to overcome in managing rare diseases. Due to the few cases, medical knowledge and expertise are limited and often insufficient. Diagnostic tests are frequently unavailable or restricted, leading to delayed, incorrect, or even missing diagnoses. Treatments are often only symptomatic, palliative, or inadequate. Access to specialist services and orphan drugs for rare disease patients is challenging, limited, and often inequitable. The lack of appropriate health policies restricts the funding allocated to this area. These challenges contribute to a loss of trust in the public health system among patients with rare diseases and their families and increase social isolation [3].

In Romania, there is a continuous concern to raise awareness of rare diseases by developing specialized medical and social services for patients and their families. This includes the use of telemedicine and artificial intelligence, which can help the sector address new challenges.

Evidence-based clinical practice in the field of rare diseases is a continuing struggle. There is a pressing need for integrated initiatives to drive research forward, particularly the creation of a coordinated clinical data management system for collecting, storing, and analyzing data from multiple diseases and clinical research centers [19]. Assessing quality of life in health research is challenging, as it requires selecting the most appropriate measures to capture the impacts of disease and treatment that are important and relevant to patients’ lives [20].

This study aimed to collect health-related data and measure the impact of rare diseases on quality of life using specific indicators such as disability level and work capacity.

Given the diverse nature of rare diseases, we assume that their effects on functionality and work capacity vary among individuals, and we want to understand and explain these variations.

To achieve this, our research followed several key steps: (a) identifying potential factors contributing to the varying impact of rare diseases; (b) providing evidence to support recommendations for rehabilitation strategies and the development of public policies, including work inclusion programs.

## 2. Methods

An observational, retrospective study was performed to evaluate persons with rare diseases admitted to the Internal Medicine Department of the National Institute of Medical Assessment and Work Capacity Rehabilitation (INEMRCM, the Romanian abbreviation) over a 5-year period, trying to understand limitations and to propose appropriate interventions. Diseases were included in the analysis if they were referenced in both the Orphanet inventory and the NORD database.

Some sociodemographic data were collected, including age, sex, and residence. Medical status was described using several parameters: duration of the disease, associated pathologies, type of treatment received, etiological category of the disease, and the disease’s functional impact. Participants were also asked about vocational factors such as educational level, occupation, and years of work experience.

The type of treatment was assessed, distinguishing between those who received a specific treatment (such as replacement therapy) and those who received supportive care focused on managing symptoms and complications.

Various types of diseases were identified and categorized based on their etiology, whether idiopathic, genetic, or autoimmune. They were also classified according to their impact on an individual’s functioning, using health-related domains from the International Classification of Functioning, Disability and Health (ICF). These categories included multisystem diseases, neuromuscular/skeletal or movement-related diseases, and sensory disorders [7].

The International Standard Classification of Education (ISCED) was used as a reference for determining education levels [21]. Based on the typical educational requirements for different occupations, three main categories were analyzed: low, medium, and high, as commonly reported in EU education statistics [22]. ISCED levels 1 and 2 (primary and lower secondary education) were combined, as were ISCED levels 3 and 4 (upper secondary and postsecondary education). Tertiary education was treated as a separate category (ISCED 6 or higher).

Occupations were classified according to the Romanian version of the International Standard Classification of Occupations—ISCO, named as the Classification of Occupations in Romania (COR), using the major occupational groups. To streamline statistical analysis, these were grouped into three broad categories based on the nature of the work and the level of training required: (a) Groups 1 and 2, which include intellectual occupations; (b) Groups 3, 4, and 5, which consist of specialized jobs typically requiring an intermediate level of training; and (c) Groups 6, 7, 8, and 9, which involve manual work with a predominantly physical component [23].

Studies frequently refer to these three levels—high, medium, and low—when using occupation as a variable in the analysis, as they indicate the skill levels required for various jobs (e.g., high-level skills for professionals and basic skills for elementary occupations) and reflect individual socioeconomic status [24,25,26].

Work capacity was considered as a binary variable, depending on the remaining functional status: completely preserved or diminished. Work disability was considered on three degrees as they are specified in Romanian law [17].

Functional impairment was analyzed using a two-level severity scale: mild vs. significant. Mild functional impairment corresponds to fully preserved work capacity and 3rd-degree work disability. Significant functional impairment grouped together 1st and 2nd-degree work disability. The 3rd-degree work disability allows for more regular professional activity under normal conditions, often part-time. In contrast, the 1st and 2nd-degree categories involve significant functional impairments, with rare employment. In these cases, work arrangements typically involve contracts with reduced daily hours, flexible schedules, and frequent work from home.

### 2.1. Statistical Analysis

A descriptive analysis was made to present sample characteristics. Means and standard deviations were calculated to describe numerical variables (e.g., age, length of service duration, retirement age, years of disease evolution, or work disability), and frequencies were used to describe categorical variables (e.g., sex, residence, disease etiology, type of impairment, treatment, level of education, type of occupation). Logistic regression analysis was employed to evaluate the estimated impact of each variable as a potential predictor of work capacity. All statistical analyses were performed by PSPP.3 software. *p* < 0.05 was the cut-off for statistical significance with a 95% confidence interval.

### 2.2. Ethical Statement

Compliance with ethical norms was certified by the Ethics Committee of the National Institute for Medical Assessment and Work Capacity Rehabilitation (INEMRCM, the Romanian abbreviation) Bucharest with No. 27/19.11.2024. This study adhered to the World Medical Association’s Declaration of Helsinki. Patients have given consent to use their data at the time of admission, and the data were analyzed anonymously.

## 3. Results

### 3.1. Analysis of Sociodemographic, Medical, and Vocational Factors

#### 3.1.1. Sociodemographic Parameters

In regard to sex, we noticed a slight predominance of women, with a female/male ratio of 48/42 persons. The mean age of the group was 44.50 ± 10.61 years (min. 19; max. 64 years). There was a significantly higher number of patients from urban areas: 62 vs. 28 persons from rural areas; *p* < 0.001.

The main characteristics of patients are presented in Table 1.

#### 3.1.2. Medical Parameters

Referring to health-related domains from ICF, there were 18 cases of global (multisystem) disorders, 11 cases of skeletal or movement-related diseases, and 61 cases of visual diseases. Associated medical conditions were recorded in 48 persons. The rare diseases affecting the patients are listed in Table 2.

The mean disease course was 10.61 ± 9.76 years (min. 1; max. 40 years). The evolution of the disease and the duration of the disease at the time of retirement were significantly longer in urban areas, 12.07 ± 10.46 vs. 7.50 ± 7.27 years; *p* = 0.022, respectively, 7.90 ± 9.86 vs. 5.29 ± 6.34; *p* = 0.020.

#### 3.1.3. Work-Related Parameters

We collected data on the education levels and occupations of 80 individuals. Of these, 38 had completed up to lower secondary education, 39 had education up to the post-secondary level, and 3 had attained a university-level degree. Occupations were categorized according to the ISCO/COR classification: 3 individuals were in intellectual occupations, 20 were in specialized work, and 57 were in manual labor. The average length of service was 13.72 ± 11.87 years, ranging from a minimum of 0 to a maximum of 38 years.

Parameters associated with work disability were analyzed, including average age at retirement, duration of illness at retirement, if retirement was caused by the disease, and distribution according to functional impairment.

The average age at retirement was 41.21 ± 10.85 years, the duration of illness at retirement was 7.09 ± 8.91 years, and the disease as a factor contributing to retirement was 76 cases, representing 84.44%; *p* < 0.001.

Distribution according to work capacity and functional impairment was as follows: preserved work capacity in 11 persons (12.22%) vs. diminished work capacity with 3rd, 2nd, or 1st degree of work disability, respectively, 25, 32, and 22 persons. Referring to two-level categories of functional impairment—mild and significant—the diseases tended to lean towards significant severity, with 54 cases (60%) compared to 36 cases (40%) classified as mild (Table 3).

In the following stages, the potential impact of various factors on work experience, retirement age, functional status, and work capacity were examined.

### 3.2. Impact on Work Participation in Terms of Work Experience and Retirement Age

The type of impairment significantly reduced the length of service in musculoskeletal and visual diseases. In global vs. musculoskeletal vs. visual diseases, the length of service was, respectively, 19.18 ± 12.28/4.17 ± 6.62/13.06 ± 11.48 years; *p* = 0.020.

In men, we noticed significantly younger retirement ages: 39.10 ± 12.26 vs. 43.06 ± 9.32 in women; *p* = 0.037, and more years of disability: 4.15 ± 6.04 vs. 2.68 ± 3.85 in women; *p* = 0.017.

### 3.3. Impact on Functional Impairment and Work Capacity

Men had more severe forms of diseases; of 42 patients, 31 (73.81%) had serious impairments vs. 11 (26.19%) with mild impairments; *p* = 0.018.

The type of impairment had an impact on individual functioning as well: Those with global impairment can work more frequently: 15 out of 18 patients (83.33%) with mild impairments vs. 3 (16.67%) with serious impairments; *p* < 0.001. Those with visual impairment have more severe impairments: 45 from 61 patients (73.77%) with serious impairments vs. 16 patients (26.23%) with mild impairments.

Referring to etiology, less disabling diseases were predominant in autoimmune conditions in 6 out of 7 patients (85.71%). In contrast, genetic conditions had a more severe functional impact in 51 out of 80 patients (63.75%); *p* = 0.037.

All individuals who received specific treatment for the disease had a better functional status, unlike only 37.21% of those who received a supportive cure; *p* = 0.023.

All the results except the etiology of the disease remain consistent when work capacity is considered as a binary outcome (preserved or reduced).

Education level and occupation were not correlated with functional impairment or work capacity (NS).

In summary, several variables were associated with better functional status and greater work capacity: sex, disease etiology, type of impairment, and treatment. Conversely, some variables, such as education level and occupation, showed no impact.

Using logistic regression, we investigated the influence of each factor on work capacity. We initially introduced all variables that were significantly correlated with work capacity—specifically sex, etiology, type of impairment, and treatment. Through the backward selection process, we eliminated sex and etiology, as they lost statistical significance in the multivariate model. The R-squared value indicated that the model was reasonably effective in predicting factors associated with better work capacity (Table 4). In conclusion, the type of impairment (mainly mobility or visual impairment) and lack of specific treatment can serve as predictors of a low likelihood of employment in cases of rare diseases.

## 4. Discussion

We encountered a group of young patients, predisposed to poor evolution in the presence of some features: male sex, in the case of genetic diseases, if they have mainly neuromuscular or visual impairments, and if no specific cure is available. In this case, their functional level is significantly reduced, and their professional activity is limited, resulting in reduced work experience.

### 4.1. Sex and Rare Diseases

In our study, men had a lower retirement age and a longer duration of disability compared with women. This could be attributed to the greater severity of diseases observed in men within our group. There are various sex-related considerations regarding rare diseases, ranging from different disease expression or severity between men and women to inequalities in disease management.

Men were also found with more severe forms of diseases. It is known that some rare diseases may be more severe in men, determined by specific genetic, biological, and hormonal factors, e.g., X-linked disorders, Duchenne muscular dystrophy or imprinting disorders, Prader–Willi syndrome.

Other studies reported poorer general health status in males than in females. This was found for patients with Fabry disease, where females are heterozygous for the lysosomal enzyme agalactosidase A (*GLA*) mutation or experience X-chromosome inactivation, and they may be asymptomatic or have only mild symptoms [27,28].

Another condition is X-linked retinitis pigmentosa (XLRP), which primarily affects males. Females who are heterozygous for an XLRP mutation are often considered unaffected carriers and may be asymptomatic, although some females may have significant visual impairment, a phenomenon related to variable patterns of X chromosome inactivation. Also, the rate of disease progression in men with XLRP is significantly higher than that in women with XLRP [29] and should be closely monitored.

Sex can be a major prognostic factor for clinical manifestations and disease evolution, as it is cited for more visual decline associated with optic glioma in neurofibromatosis type 1 for females and more cognitive deficits in males [30]. Women tended to display considerably less severe cardiomyopathy in familial amyloidosis [31].

Men with retinitis pigmentosa had higher mortality due to suicide, which was associated with poor mental health and depression resulting from the disease and its unfavorable prognosis [32]. Romanian society is traditionally structured, with men being taught from a young age that their primary role is to be the pillars of the family. Research has shown that men’s loss of this traditional role, particularly as breadwinners, can have a negative impact on the family as a whole. This is especially true if the man has strongly developed a leadership mindset and struggles emotionally and behaviorally to cope with the challenges of being diagnosed with a serious illness and the resulting functional limitations [33]. Emotional and psychological effects may include anxiety about further functional loss, depression, and difficulties adjusting to changes in self-image and autonomy.

Autoimmune diseases were more prevalent in women, whereas the gender difference was less pronounced for genetic conditions, and idiopathic diseases were observed exclusively in men. This distribution likely plays a role in the better prognosis observed in women, as targeted therapies are available for autoimmune diseases.

Although statistical significance was not reached for certain parameters (such as disease duration) these aspects warrant further investigation in larger patient cohorts. In contrast, the significantly lower retirement age among men may be attributed to the more severe progression of the diseases in this group. Additionally, various factors—both individual and related to social and professional environment—could influence one’s ability to work, with the type of work potentially playing a contributing role.

Differences in disease management between the sexes may also exist, considering that men had significantly longer disease duration and extended periods of work disability. Women who often engaged in domestic work may have delayed seeking medical evaluations and claiming social insurance rights.

Several European studies have shown gender disparities alongside other factors—socioeconomic, geographical and cultural—that create barriers to access to care. From diagnosis to access to treatment or care responsibilities, women face several challenges that constitute an important but underestimated threat to their health and quality of life. Similar observations were reported for rare diseases, where gender inequalities overlap with the rarity, complexity, degenerative and often life-threatening characteristics associated with these conditions. It is crucial to implement solutions that guarantee access to care, opportunities, and rights in the labor market without any form of discrimination, including sex-based discrimination. More research is needed to fully understand and address all facets of gender inequality in both healthcare and the economy.

### 4.2. Etiology and Rare Diseases

The etiology of the disease might be linked to the degree of functional impairment. The literature shows that more than 70% of rare diseases are of genetic origin [34]. The impact of a rare disease is influenced by its genetic basis, the age of onset, the body systems it affects, and the extent to which it impacts the individual’s quality of life, functional capacity, and overall health.

Autoimmune diseases or conditions with specific treatments generally showed better progression, with patients preserving greater functional status. This improvement contributes to a higher quality of life and a greater likelihood of being able to engage in professional activities. In contrast, genetic disorders or diseases without specific treatments—where therapy mainly focuses on symptom management or addressing complications—tended to have poorer outcomes, with substantial declines in overall function. The advantages of early genetic testing before birth become evident. Early diagnosis of genetic conditions enables parents to make informed decisions about the pregnancy, facilitates early interventions (including surgical treatments), and allows them to prepare emotionally, logistically, and financially for the care their child may require after birth.

### 4.3. Disability in Rare Diseases

The diseases had a significant and negative impact on the patients’ professional situations. Seventy-six cases (84.44%) retired because of the disease; a significantly higher percentage compared to the 50.7% reported in the French barometer survey [35]. They had an average retirement age of 41 years, which was substantially lower than the standard retirement age of 65. Additionally, their employment duration was lower than the minimum statutory requirement of 15 years [17].

In our study, a significant proportion of 60% (54 cases) were classified as having more disabling diseases. These conditions were associated with increased functional impairment and greater adaptive needs for maintaining professional activities. Similar findings have been reported in the literature, although typically focusing on a single pathology. Nätterlund has noted a significantly reduced functional capacity in more than one-third of individuals with muscular dystrophy [36]. Speaking in terms of work ability, the estimated work ability in our group (full or part-time) of 40%, was less than the 55.1% reported by Velvin in a scoping review on work participation in adults with rare genetic diseases, or the employment rate of 49% communicated by Heuyer in the French barometer survey from 2015 [12,35].

We have carried out a complex analysis, considering the various ways in which work can be performed and insurance benefits accessed across Europe. This approach aimed to capture a broad range of situations while importantly considering the preference of disabled workers for part-time employment. This option is often viewed as beneficial, as it enables individuals to achieve a better balance between their personal and health needs and their professional lives, as highlighted by other researchers. The European analysis conducted by Pagan R. revealed that the percentage of part-time employment was higher among people with disabilities compared to those without disabilities in nearly all the countries examined [37].

The type of functional impairment had a long-term role in our group. This feature is in line with the findings of a meta-analysis conducted on the economic impact of rare diseases by Sequeira et al. They reported that patients with musculoskeletal diseases seemed to have the lowest quality of life, while autoimmune disorders seemed to have better outcomes [38].

Neurological rare diseases like Duchenne muscular dystrophy (DMD) and physical disabilities caused by other conditions like osteogenesis imperfecta (OI) are generally more disabling over time compared to many other rare diseases. These conditions tend to have a progressive course, tending to worsen as the disease progresses, significantly impacting the individual’s quality of life and functional independence. For this category, mobility devices can be beneficial and should be readily accessible.

The severe limitation was reported by other authors in diseases with skeletal involvement, e.g., in individuals with osteogenesis imperfecta (OI) type III who experience recurrent fractures and periods of immobilization, with consequent muscle weakness and deconditioning, which leads to more expressive functional limitations [39].

Steinert myotonic dystrophy, a hereditary chronic systemic disease, is characterized by muscular weakness, often involving loss of the ability to walk and increased dependence on others and the need for technical aids. Compared to other neuromuscular diseases, persons with myotonic dystrophy were found with more functional disabilities and higher dependence on others for activities of daily living.

Also, general health scores may be poorer in some diseases, for example, as was reported for Fabry disease when compared to Gaucher disease patients [40].

Diseases that affect vision cause significant disability. X-linked retinitis pigmentosa (XLRP) is a rare, incurable, progressive genetic disease that causes patients to lose independence over time, often becoming blind by the age of 45 and affecting their participation in the workplace. Qualitative studies have described for people living with RP difficulties in performing daily tasks and barriers to work and career. People have also described the emotional impact of coping with the progression of RP, ongoing social and physical challenges, and the impact of RP on relationships.

The economic burden of these diseases is associated with lost productivity, higher healthcare costs, and increased demands for formal and informal care [41]. Qualitative studies have described difficulties in employment, reduced job opportunities, and consequently, a financial impact for people with RP. Difficulties navigating unfamiliar or crowded environments have led to occupational restrictions and sometimes a requirement to change jobs as visual acuity decreased [29].

Patients were significantly more likely to come from urban areas. One possible explanation could be that individuals in rural areas have limited access to diagnosis and treatment. Numerous studies have demonstrated that social determinants of health are strongly connected to disparities in health outcomes. Individuals in lower socioeconomic groups tend to face later diagnoses, more advanced stages of the disease, shorter life expectancy, and overall poorer health [42]. The availability of support services and specialized healthcare infrastructure can significantly improve management and outcomes. These can include access to healthcare specialists (e.g., geneticists, neurologists, cardiologists) and rehabilitation—physical therapy that can help improve functional outcomes, particularly in diseases with muscle weakness or mobility impairments.

### 4.4. Effect of Treatment in Rare Diseases

In our study, patients with diseases with specific treatments had better outcomes. The availability of effective treatments is a critical factor, but unfortunately, for many rare diseases, treatment options are experimental or non-existent. This lack of specific therapies can lead to poor prognosis and higher morbidity. Targeted treatments for specific diseases (e.g., enzyme replacement therapy for Fabry or Gaucher disease) have significantly improved outcomes for these conditions and extended life expectancy, but these are not frequent. In the absence of curative treatments, therapies generally focus on managing symptoms and preventing complications, thus improving quality of life (QoL) [43,44,45,46,47]. Multidisciplinary care and psychosocial support can also help reduce the impact of the disease on daily life and potentially extend life expectancy by improving the overall health and well-being of the individual.

Despite the fact that about 95% of rare diseases are still without specific treatments, the intervention has been directed to control symptoms and improve QoL; studies focused on specific treatment showed great improvements in physical and mental health, the ability to perform work and daily activities [38,48,49].

Treatment was described as effective by Weinreb et al. (2007), who evaluated enzyme replacement therapy (ERT) efficacy after 4 years of treatment and reported a positive effect on the QoL of patients with Gaucher disease [50]. Improvements in several outcomes, clinical improvement, functional independence and QoL were observed with ERT in Fabry disease, for both women and men [51,52,53]. This underscores the critical need for efforts to develop targeted treatments for rare diseases, which could greatly improve patient outcomes.

Strength of the study. The heterogeneity of diseases, characterized by diverse causes and varied manifestations, has significantly broadened our understanding of their potential evolution and impact on people’s lives. The medical, and particularly the social perspective, offers a less familiar yet more comprehensive framework for understanding the impact of these diseases. It addresses the complex needs of this category of patients, enabling more targeted and effective interventions for rare conditions, which often present unique challenges. Few articles focus on work participation in rare diseases, particularly as a primary outcome, and most of these studies examine a single disease. Identifying some key factors associated with poor functional status over time is another strength of our study. This can help predict employability trajectories and potentially enable early interventions with favorable outcomes.

Study limitations. The small number of patients in this study limits the generalizability of the findings. This study did not analyze the patients’ perspectives or how families cope with illnesses and their consequences, with valuable missing data. A strong support system, mainly family and caregivers, is crucial for mitigating the challenges of living with a rare disease. A lack of social support can increase the burden of caregiving, exacerbate psychological stress, and hinder the patient’s ability to function independently. Social isolation, which is common in rare disease patients due to the rarity of their conditions and the stigma they may experience, can significantly affect mental health and functional status. Also, environmental factors were not studied. Living conditions and accessibility, in addition to education, can also influence functional outcomes, particularly when mobility aids or other adaptive devices are needed. Lack of access to healthcare resources can delay diagnosis, limit treatment options, and hinder access to supportive services. Financial constraints due to the cost of treatment, caregiving, and the inability to work, can negatively impact functional outcomes.

Further directions for research. Given the limitations of the current study, there are several key directions for future research that could address the gaps identified and contribute to a more comprehensive understanding of the impact of rare diseases on patients and their families.

Cooperation with rare disease centers or patient associations could facilitate the inclusion of more patients with diverse rare diseases in the future, enhancing statistical power and ensuring the findings better represent the broader rare disease community. Moreover, participating in clinical trials and joining an online community can aid in managing life with a rare disease. Follow-up studies would offer valuable insights into how patients experience their disease over time, enabling healthcare providers, researchers, and policymakers to better understand the true long-term effects and needs of rare disease patients.

Collecting data on patients’ experiences of living with a rare disease, their psychosocial well-being, and quality of life allows for tailored care plans adapted to their needs and values that address not only clinical symptoms but also the psychological and social dimensions of the illness. Understanding the family role and stress and the strategies families use to manage the challenges could help develop interventions to support families and improve overall outcomes for patients [54].

Greater multidisciplinary collaboration is mandatory to develop good practices for this category of patients. Because a rare disease affects every aspect of a person’s daily life, including work and home life, it is important to extend assessment to real-life experience to improve our understanding of the natural course of diseases and guide treatment outcomes.

## 5. Conclusions

Patients with rare diseases are very heterogeneous in terms of functional capabilities, so in most situations, case-to-case analysis is mandatory for framing different categories of work or medical retirement. Sex, disease etiology, and, in particular, functional expression and type of treatment are potential factors that can influence care results. Among these, the type of impairment and the availability of specific treatments might be predictors of a higher probability of employment. These factors may impact disease progression, personal functioning, and the ability to maintain specific roles within family and society. Higher functional capacity enhances the quality of life in multiple ways: it promotes greater independence by enabling individuals to perform daily activities, facilitates engagement in social activities, supports workforce participation, and subsequently increases self-esteem, social interactions, and financial stability.

Our study indicates the need to broaden research strategies for rare diseases, incorporating additional factors like physiological and environmental exposures to create a more tailored intervention profile. Additionally, further studies on larger groups are needed to enable more comprehensive analysis and increase reliability. This kind of approach is challenging because of the scarcity of cases (the rare diseases are… rare), and it may require transnational studies. Effectively managing the unique characteristics within the diverse manifestations of these diseases—using well-known and widely adopted tools while considering varying regulations and social contexts across countries—could be crucial for improving patient outcomes.

## Figures and Tables

**Table 1:** Main sociodemographic, medical, and vocational characteristics of participants.

Characteristics	Values ± SD/Number (%)
Mean age (years)	44.50 ± 10.61
Sex (female)	48 (53.33%)
Residence (urban)	62 (68.89%)
Mean disease duration (years)	10.61 ± 9.76
Comorbidities (yes)	51 (56.67%)
Treatment (specific)	4 (4.44%)
Mean of work experience (years)	13.72 ± 11.87

**Table 2:** Overview of the cases.

Etiology of the Diseases (Nr. of Cases)	Diseases Included (Nr. of Cases)
Idiopathic (3)	Madelung’s disease/Benign Symmetric Lipomatosis (1)
	Legg–Calve–Perthes disease (1)
	Paget’s disease of bone/Osteitis Deformans (1)
2. Genetic (80)	Hereditary amyloidosis (1)
	Bourneville disease/Tuberous sclerosis complex (1)
	Charcot–Marie–Tooth disease (2)
	Darier’s disease/Dyskeratosis follicularis (1)
	Fabry disease/Alpha-galactosidase A deficiency (2)
	Gaucher disease/Glucocerebrosidase deficiency (1)
	Hypogammaglobulinemia (1)
	Klippel–Feil syndrome (1)
	Laurence–Moon syndrome/Adipogenital-retinitis pigmentosa syndrome (1)
	Morris syndrome/Androgen insensitivity syndrome (1)
	Osteogenesis imperfecta (1)
	Prader–Willi syndrome (1)
	Recklinghausen’s disease/Neurofibromatosis type 1 (5)
	Retinitis pigmentosa (39)
	Stargardt disease/Fundus flavimaculatus (12)
	Steinert myotonic dystrophy (1)
	Usher syndrome/Retinitis pigmentosa-deafness syndrome (8)
	Wilson’s disease/Hepatolenticular degeneration (1)
3. Autoimmune (7)	Adult-onset Still’s disease (1)
	Sharp syndrome/Mixed connective tissue disease (1)
	Achalasia (1)
	Castleman disease/Angiofollicular lymph hyperplasia (1)
	Duhring–Brocq disease/Dermatitis Herpetiformis (1)
	Devic’s disease/Optic neuromyelitis (1)
	Myasthenia gravis (1)
TOTAL	90 cases

**Table 3:** Distribution according to work ability and functional impairment.

Cases (90)	Work Ability (No of Cases/%)	Work Disability	Functional Impairment (No of Cases/%)
11	Preserved 11 (12.22%)	Absent	Mild 36 (40%)
25	Reduced 79 (87.78%)	3rd degree	
32		2nd degree	Significant 54 (60%)
22		1st degree	

**Table 4:** Logistic regression analysis with predictive factors for employment (R squared = 0.25).

Variable	B Coefficient	Standard Error	Significance (*p*)
Constant	26.70	3.58	<0.001
Treatment	−28.64	3.35	<0.001
Type of impairment	2.16	0.53	<0.001

## Data Availability

Data are contained within the article.
